# Mapping and Functional Characterisation of a CTCF-Dependent Insulator Element at the 3′ Border of the Murine Scl Transcriptional Domain

**DOI:** 10.1371/journal.pone.0031484

**Published:** 2012-03-01

**Authors:** George A. Follows, Rita Ferreira, Mary E. Janes, Dominik Spensberger, Francesco Cambuli, Amy F. Chaney, Sarah J. Kinston, Josette R. Landry, Anthony R. Green, Berthold Göttgens

**Affiliations:** Department of Haematology, Cambridge Institute for Medical Research, University of Cambridge, Cambridge, United Kingdom; University of Birmingham, United Kingdom

## Abstract

The Scl gene encodes a transcription factor essential for haematopoietic development. Scl transcription is regulated by a panel of cis-elements spread over 55 kb with the most distal 3′ element being located downstream of the neighbouring gene Map17, which is co-regulated with Scl in haematopoietic cells. The Scl/Map17 domain is flanked upstream by the ubiquitously expressed Sil gene and downstream by a cluster of Cyp genes active in liver, but the mechanisms responsible for delineating the domain boundaries remain unclear. Here we report identification of a DNaseI hypersensitive site at the 3′ end of the Scl/Map17 domain and 45 kb downstream of the Scl transcription start site. This element is located at the boundary of active and inactive chromatin, does not function as a classical tissue-specific enhancer, binds CTCF and is both necessary and sufficient for insulator function in haematopoietic cells *in vitro*. Moreover, in a transgenic reporter assay, tissue-specific expression of the Scl promoter in brain was increased by incorporation of 350 bp flanking fragments from the +45 element. Our data suggests that the +45 region functions as a boundary element that separates the Scl/Map17 and Cyp transcriptional domains, and raise the possibility that this element may be useful for improving tissue-specific expression of transgenic constructs.

## Introduction

The Stem Cell Leukemia (Scl) gene, also known as Tal1, encodes a basic helix-loop-helix transcription factor with important roles in the development of the haematopoietic [1]–[3] vascular [4], [5] and central nervous systems [4], [6], [7]. Ectopic expression of Scl is detrimental for the normal differentiation of the affected lineages. For example, SCL overexpression in T-cells is one of the most common molecular abnormalities found in T-ALL [8]. Appropriate spatio-temporal transcription of Scl is therefore essential for the normal development and homeostasis of the haematopoietic system and this focuses attention on the mechanisms controlling Scl transcription.

In order to understand the mechanisms involved in the regulation of Scl expression, we have systematically dissected the cis-regulatory elements operating at the murine Scl locus. Comparisons of Scl flanking genes during vertebrate evolution revealed a limited region of conserved synteny, spanning the Scl and Map17 genes, likely to contain regulatory elements responsible for the conserved pattern of Scl expression [9], [10]. A 100 kb DNA fragment encompassing the entire Scl/Map17 domain is sufficient to reproduce endogenous Scl expression and rescue the Scl knockout phenotype [11]. The Map17 gene is expressed in skin, liver and kidney and is co-regulated with Scl in haematopoietic cells [12]. Studies of the chromatin structure of the mouse Scl/Map17 locus, combined with comparative sequence analysis and reporter assays in transgenic mice have allowed us to characterize the Scl and Map17 promoters together with a panel of enhancers [13]. The Scl promoters have no hematopoietic activity and drive Scl expression in the central nervous system [14]. Haematopoietic-specific expression is achieved due to the presence of the tissue-specific enhancers −4, +19 and +40 (named in kb with respect to the start of exon 1a). Each of these elements directs expression to a subdomain of the normal Scl expression pattern in transgenic mice [9], [10], [12], [14]–[19]. In mammals the Scl/Map17 co-regulatory domain is flanked upstream by the ubiquitously expressed Sil gene and downstream by a battery of cytochrome genes mostly expressed in liver, however the Cyp gene immediately downstream, Cyp4x, is highly expressed in brain [20]. This arrangement suggests the existence of boundary mechanisms that insulate the Scl/Map17 domain from its neighbours. In this paper we characterize a novel DNase I hypersensitive site (+45 element) at the telomeric end of the Scl/Map17 domain, and demonstrate that it functions as a CTCF-dependent insulator both *in vitro* and *in vivo*.

## Methods

### DNaseI hypersensitive site (HSS) mapping

Briefly, nuclei from 416B, mES cells and primary mice thymocytes were isolated and treated with DNaseI. DNA from treated nuclei and genomic DNA (Input) was isolated, labelled with Cy3/Cy5 and hybridised to DNA microarrays as previously described [21], [22]. The Scl DNA microarrays were synthesised by FlyChiP using PCR amplicons of non-repeat regions with an average size of 500 bp as previously described. Details of PCR amplicons are available on request. Following hybridisation, slides were analysed as previously described.

### Luciferase assays

416B cells were co-transfected with the constructs described bellow and the PGK-Neo construct and selected for Neo resistance. Luciferase assays were performed on selected cells as described previously [19]. The basic constructs SV/Luc, Map17/Luc and Scl/Luc have all been used in transfection assays previously [15], [19]. The +45 element was mapped from the HSS experiments as 1.76 kb fragment (genomic co-ordinates, chr4: 114773516–114775283). This element was amplified by PCR and cloned using specific restriction sites. The 350 bp was flanked by an NsiI and EcoRI site (located at chr4: 114774278–114774628), and these sites were used for the generation of Scl/Luc/350 construct and deletion of the 350 region from Scl/Luc/+45 (Scl/Luc/del350) using standard cloning techniques. All constructs were sequence verified.

### Chromatin Immunoprecipitation (ChIP)

The ChIP assays were performed as previously described [12]. Briefly, protein-DNA complexes were cross-linked using 0.4% formaldehyde followed by cell lysis. Sonication of DNA was performed using Bioruptor sonicator (Diagenode). The antibodies used were anti-acetylated histone 3 lysine 9 (Upstate, Millipore), anti-trimethylated histone 3 lysine 27 (Upstate, Milipore) and anti-CTCF (Upstate, Millipore).

### Microarray hybridization

Hybridization of DNAse1 HSS and ChIP samples to Scl tiling path microarrays as been described before [21]. Briefly, samples were labelled with Cy3 and/or Cy5, pooled and hybridized to the Scl tiling path microarrays using an automated TECAM 400 hybridization station and scanned using a ScanArray 4000 confocal laser-based scanner (Perkin Elmer). Mean spot intensities were quantified using ScanArray Express (Perkin Elmer) with background subtraction. Mean spot intensities from replicate samples were use to generate mean value from each data point. Data is presented as Cy3/Cy5 ration, where Cy3 represents the sample and Cy5 the input.

### *In vivo* dimethylsulphate (DMS) footprinting

For *in vivo* DMS footprinting experiment we performed ligation-mediated PCR techniques previously published [23]. Briefly, cells were treated with 0.2% dimethyl sulfate (DMS) in PBS before DNA extraction and piperidine treatment as described in [24]. Primer sequences are available upon request.

### Electrophoretic Mobility Shift Assay (EMSA)

EMSA was performed using standard techniques. The probe DNA sequence used was the 57 base pair sequence identified from the DMS footprinting experiments. The probe was end-labeled using polynucleotide kinase, 30 pmol of oligos, and 2 µCi [γ-32P] ATP. The cold probe of either wild type sequence or mutated sequence was prepared as standard probe with the exclusion of 2 µCi [γ-32P] ATP. The cold probe was added in 10× excess as compared to the hot probe. For supershift the nuclear extract with probe was incubated with 50 ng of specific CTCF antibody (Upstate, Millipore).

### Insulator Assays

Insulator transfection assays were performed in K562 cells as described previously [25]. Transfected cells were plated onto a methylcellulose (StemCell Technology) base medium supplemented with 650 µg/ml of G418 (Invitrogen). All of the DNA fragments were cloned between the enhancer and the neomycin resistance gene in the pNI vector. The 1.2 kb fragment containing the chicken hypersensitive site −4 insulator (cHSIV- genBank #U78775)) was used as the positive control as previously published [26]. The −8/−9 region, the +45 and the +45del350 were amplified from 416B genomic DNA and cloned into pNI vector. The +45del350 construct was generated by subcloning 350 bp, from the +45 fragment, into the pNI vector using NsiI and EcoRI restriction sites. The 350-del57FPR construct was generated by deleting 57 base pairs of the footprinted region. For the 57 bp footprinted region (57FPR) construct the fragment was amplified by PCR and cloned into pNI expression vector. The 350MutCTCF construct was generated using a direct mutagenesis kit (Stratagene).

### Transgenic reporter assays

F0 transgenic reporter assays were performed using techniques previously reported [12]. Briefly, oocytes were injected with linearized constructs and embryos were harvested at embryonic day (E) 12.5. Embryos were stained with 5-bromo-4-chloro-3-indolyl-ß-D-galactopyranoside (X-Gal) for the detection of LacZ expression as described previously. The SV40-LacZ and the Scl-LacZ constructs were generated and used previously in transgenic reporter assays [15]. The +45, +45 del350, and the 350 elements were subcloned from pNI vector into the pGL2 expression vector [12]. Genotyping was performed using specific primers for LacZ gene, available on request. The staining pattern was defined by microscopic inspection of the embryos. All animal work as been conduct in agreement with the United Kingdom Home Office regulations under the “Animals (scientific procedures) act 1986” project licence number PPL 80/2376.

## Results

### The Scl/Map17 co-regulatory domain is bounded by a DNase I hypersensitive site 45 kb downstream of the mouse Scl promoter 1a

**Figure 1:**
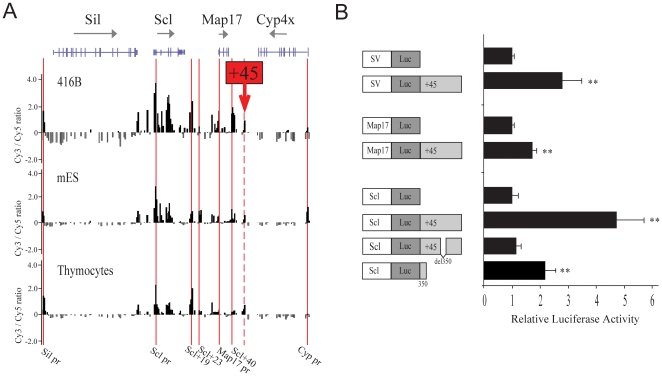
Identification and functional analysis of a novel 1.76 kb Scl element located 45 kb downstream from Scl promoter. (**A**) DNaseI hypersensitive profiles across 130 kb (NCBI37/mm9 chr4:114,670,057–114,799,426) of the extended Scl locus in 3 cell types: mouse haematopoietic progenitors (416B), mouse embryonic stem cells (mES) and primary mouse thymocytes. The location of the 4 genes at the locus are represented at the top of the figure (Sil, Scl, Map17 and Cyp4x) with direction of transcription indicated by the arrows and translated and untranslated exons represented by wide and narrow bars respectively. The location of known key regulatory elements is indicated by the red bars (Sil promoter, Scl promoter, Scl+19, Scl+23, Map17 promoter, Scl+40, and Cyp promoter), and the location of the putative +45 element is indicated with a dashed red line. The data presented is a representative experiment showing fold enrichment over non-enriched input plotted (log_2_) against genomic position. (**B**) Luciferase activity is increased in 416B cells stably transfected with constructs containing the +45 element (SV/Luc/+45, Map17/Luc/+45 and Scl/Luc/+45) when compared to promoter alone. The 350 bp core region of the +45 element (Scl/Luc/+45del350) is necessary but not sufficient for full transcriptional enhancement from the Scl/Luc/+45 construct. The left panel represents the constructs diagrammatically. Luciferase activity was calculated as fold increase over the promoter only construct. The data representing mean +/− SD relative luciferase activity from 5 independent experiments, each performed in triplicate. ** indicated p<0.01 compared to promoter alone.

We have previously described and validated a high throughput array-based approach to DNaseI hypersensitive site (HSS) mapping (ADHM) that uses tiled microarrays to map HSS over large genomic distances [21]. Using this approach we characterized the DNaseI HSS profile across the Scl/Map17 locus in 416B cells (a mouse haematopoietic progenitor cell line), mouse embryonic stem cells (mES), and primary mouse thymocytes (Figure 1A). The 416B cells express Sil, Scl and Map17 but not Cyp4x, while mES and thymocytes express Sil, low levels of Map17, but not Scl or Cyp4x (Figure S1A in Information S1). A DNaseI HSS is present at the Sil promoter in each of the 3 cell types, consistent with the ubiquitous expression of this gene. The central Scl/Map17 co-regulatory domain is prominently accessible in 416B cells, with clear peaks of accessibility at previously described regulatory elements of the locus, including the +19 and +40 Scl haematopoietic enhancers. There is lower level accessibility across this domain in both mES and thymocytes which do not express Scl, although increased accessibility was noted at known regulatory elements, such as the +23 neuronal enhancer in mES cells and +19 enhancer in thymocytes. In all 3 cell types, there is minimal accessibility over the Cyp4x gene, with the exception of the promoter region, which remains accessible in mES cells.

A striking feature of the DNase I profiles from the 3 cell types is the presence of a 55 kb region of DNase I accessibility spanning the Scl/Map17 genes. At its 3′ end, the extent of this domain is marked by a DNaseI HSS 45 kb downstream of the Scl promoter 1a (+45 element). This +45 element is DNase I accessible in all cell types studied, and marks a boundary between the Scl and Map17 genes, which are expressed in developing haematopoietic stem and progenitor cells, and the Cyp family of genes, which are not. Analysis of publicly available DNaseI HSS data from the ENCODE consortium [27] using the UCSC genome browser [28] shows that the DNaseI HSS site located +45 kb downstream of the Scl promoter is not haematopoietic specific, but instead appears to be ubiquitous since it can be observed in a wide range of mouse tissues including brain, heart and liver (Figure S1B in Information S1).

### The +45 element enhances transcription from a promoter, but does not direct tissue-specific expression

To investigate whether the +45 region functions as a transcriptional enhancer, luciferase reporter constructs were generated using a 1.76 kb genomic fragment encompassing the complete +45 element and their activity was assessed in 416B cells hematopoietic progenitor cells (Figure 1B). The presence of the +45 element significantly increased luciferase activity not only with the SV40 promoter but also with the endogenous Scl and, to a lesser extent, the Map17 promoter, indicating that the enhancing activity of the +45 element is not promoter-specific. Previous genomic sequence alignments had not identified a peak of homology corresponding to the +45 element. However, manual alignment of this region revealed a 350 bp fragment with significant mouse/human sequence conservation (Figure S2 in Information S1), and deletion of this 350 bp core sequence abolished enhancer activity of the +45 element (Figure 1B). This 350 bp core region alone has enhancing activity in luciferase assays, although not as strong as the full +45 element. In order to analyze whether the +45 element functions as a cell-type specific enhancer *in vivo*, we next studied different constructs containing the LacZ reporter gene under the control of the SV40 promoter (SV/LacZ) using transgenic mice reporter assays. The SV40 promoter does not confer tissue-specific expression of the reporter gene but variable ectopic expression can be observed in F0 transgenic embryos. We have previously used this approach to characterize a range of Scl regulatory elements that direct Scl expression to specific developmental domains [10], [12], [15], [17], [19]. Constructs containing the element of interest and a SV40P-LacZ reported cassette were injected in oocytes and F0 embryos were analysed for LacZ expression at embryonic day (E) 12.5. In the presence of the +45 element the number of embryos ectopically expressing LacZ increased significantly (from 24% of transgenic embryos to 54%, p = 0.04) when compared to SV/LacZ alone, but no consistent tissue-specific expression was observed. No difference was observed between SV/LacZ and SV/LacZ/+45del350 indicating that the 350 bp core sequence is essential for the enhancing activity (Table 1). These data raised the possibility that the +45 element may not function as a tissue-specific transcriptional enhancer *in vivo*.

**Table 1:** The 350 bp core region of the +45 element insulates transgenic constructs and improves tissue-specific expression from the Scl promoter.

Construct	Total transgenic embryos	LacZ^+^ transgenic embryos		Expression pattern	Embryos with specific LacZ expression	
	(n)	(n)	(%)		(n)	(%)
Sv/Lac	21	5	24	Ectopic	0	0
Sv/Lac/+45	13	7*	54	Ectopic	0	0
Sv/Lac/+45del350	14	3	21	Ectopic	0	0
Scl/Lac	13	5	38	Brain+Ectopic	1	20
350/Scl/Lac/350	12	8	66	Brain	8**	100

The total number of F0 transgenic embryos obtained with each construct was determined by PCR using specific primers. Upon ß-galactosidase staining, transgenic embryos were visually inspected and scored as either LacZ+ or LacZ- and the percentage of LacZ+ embryos calculated.

* p<0.05 compared to SV/LacZ. The expression pattern of Scl/LacZ and 350/Scl/LacZ/350 transgenic embryos is considered ectopic if beyond the mid and hind brain, the expected expression pattern for the Scl promoter alone.

** p<0.01 compared to Scl/LacZ.

### The +45 element does not carry the histone marks of a transcriptional enhancer and is located at the boundary of the Scl/Map17 genomic region that displays active and repressive histone marks

**Figure 2:**
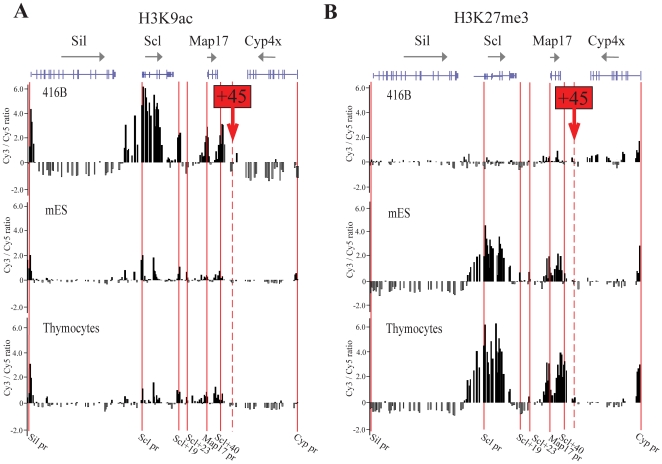
ChIP-chip profiles mapping of H3K9ac (A) and H3K27me3 (B) across 150 kb of the extended Scl locus in 3 cell types, 416B (mouse haematopoietic progenitor cells), mouse embryonic stem cells (mES) and primary mouse thymocytes. The location of the 4 genes at the locus are represented at the top of the figure (Sil, Scl, Map17 and Cyp4x) with direction of transcription indicated by the arrows and translated and untranslated exons represented by wide and narrow bars respectively. The location of known key regulatory elements is indicated by the red bars (Sil promoter, Scl promoter, Scl+19, Scl+23, Map17 promoter,Scl+40 and Cyp promoter), and the location of the putative +45 element is indicated with a dashed red line. Representative experiments showing fold enrichment over non-enriched input plotted (log_2_) against genomic position. In the cell types studied, the +45 element is located at a boundary between chromatin domains carrying transcriptionally active and/or repressive histone marks, while the H3 proteins at the +45 element itself are neither acetylated at H3K9 nor methylated at H3K27.

To gain further insights into the function of the +45 element, we investigated the presence of active and repressive histone marks across the Scl/Map17 co-regulatory domain using chromatin immunoprecipitation (ChIP; Figure 2). In 416B cells, which express high levels of Scl and Map17, there were prominent marks of histone 3 lysine 9 acetylation (H3K9ac) over known active promoters and enhancers, with lower levels of acetylation over the same regions in ES cells and thymocytes. The three prominent peaks of acetylation over the 3′ end of the Scl/Map17 co-regulatory domain in 416B cells correspond to previously characterized functional *cis*-elements, the +19 enhancer, the Map17 promoter (+32) and the +40 enhancer. In contrast, the +45 element itself did not display H3K9ac in any of the three cell types studied. To examine the distribution of repressive histone marks, we repeated ChIP-Chip experiments using an antibody to trimethylated histone 3 lysine 27 (H3K27me3, Figure 2B). In 416B cells, H3K27me3 was not detected over the Scl and Map17 genes, which are both expressed in these cells. However, in mES and thymocytes, which lack expression of Scl, high levels of H3K27me3 were detected over this genomic region. This repressive histone modification did not extend into or beyond the +45 element. Furthermore, data from the ENCODE consortium [27] confirms the presence of monomethylated and trimethylated histone 3 lysine 4 (H3K4me1 and H3K4me3) marks over the Scl/Map17 locus but not beyond the +45 element in tissues where Scl and/or Map17 are expressed, like bone marrow and kidney (Figure S3 in Information S1). Thus, the +45 element is located at the telomeric boundary of the Scl/Map17 domain that displays an active chromatin mark in Scl/Map17 expressing cells and a repressive mark in non-expressing cells.

### The 350 bp core fragment of the +45 element contains a 57 bp footprinted region that binds CTCF *in vitro* and *in vivo*

**Figure 3:**
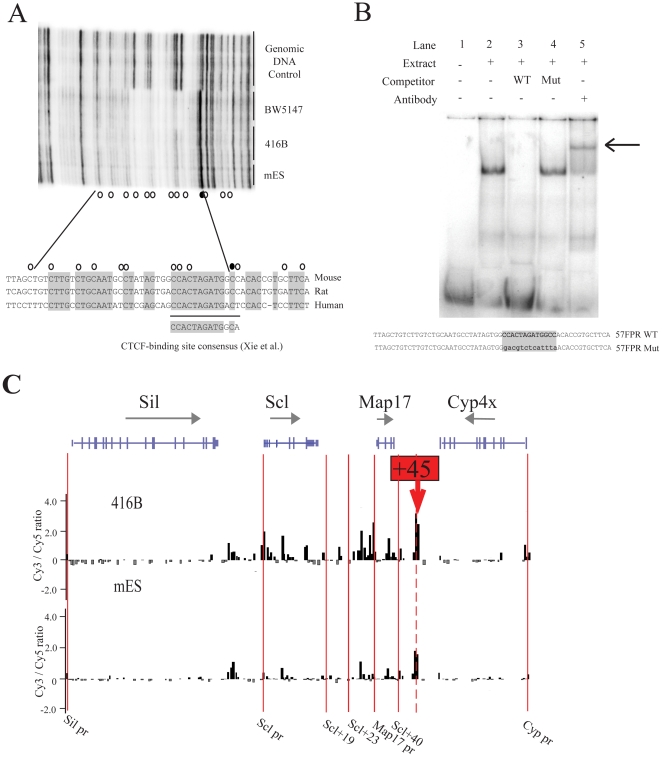
*In vivo* binding of CTCF to 57FPR sequence in haematopoietic progenitor cells. (**A**) DMS footprinting of the 350 base pair core region of the +45 element in 3 cell types, BW5147, 416B and mouse ES cells, shows a DNA protein footprint that extends to 57 base pairs in length. Open circles represent relative protection from DMS-induced methylation compared with naked genomic DNA control, while the closed circle represents enhancement of DMS-induced methylation. Multiple lanes correspond to different biological replicates. The sequence of the 57 bp footprinted region (57FPR) is shown with cross-species sequence comparison. The CTCF-consensus binding site from Xie et al is also represented. (**B**) EMSA demonstrating a direct interaction between the 57FPR and CTCF protein (lane 2). The interaction is lost by competition with wild type 57FPR (lane 3), but not by CTCF-mutated 57FPR (lane 4). The arrow indicates the super shifted complex by co-incubation with specific anti-CTCF antibody (lane 5). The wild type and mutated sequence used for the EMSA are also shown. (**C**). ChIP-chip profiles mapping CTCF binding across 150 kb of the extended Scl locus in 2 cell types, 416B (mouse haematopoietic progenitor cells) and mouse embryonic stem cells (mES). The location of the 4 genes at the locus are represented at the top of the figure (Sil, Scl, Map17 and Cyp4x) with direction of transcription indicated by the arrows and translated and untranslated exons represented by wide and narrow bars respectively. The location of certain known key regulatory elements is indicated by the red bars (Sil promoter, Scl promoter, Scl+19, Scl+23, Map17 promoter, Scl+40 and Cyp promoter), and the location of the putative +45 element is indicated with a dashed red line. Representative experiment is showing fold enrichment over non-enriched input plotted (log_2_) against genomic position. In the cell types studied, the +45 element is the most highly enriched site for CTCF across the extended Scl locus.

*In vivo* dimethylsulphate (DMS) footprinting was used to map DNA-protein interactions across the 350 bp core of the +45 element in 3 different cell types at single nucleotide resolution (Figure 3A). Samples from BW5147 cells, a T-lymphocyte cell line that does not express Scl, Map17 or Cyp4X1 [12], 416B cells and mES cells each showed a prominent 57 bp footprint when compared to the genomic DNA control. Despite very different expression levels of Scl and Map17 in the 3 cell types (Figure S1A in Information S1 and reference [12]), the pattern of *in vivo* footprinting at this element was virtually identical, and therefore concordant with the ubiquitous nature of the DNaseI HSS. The footprinted region showed a high level of sequence conservation between mouse, rat and human, and contained a core sequence highly similar to a recently published CTCF consensus sequence [29], [30].

To investigate whether CTCF binding is responsible for the footprinting results observed in Figure 3A, we performed electrophoretic mobility shift assay (EMSA) and ChIP-on-chip experiments. Using the 57 base pair footprinted region (57FPR) as the target oligonucleotide, addition of nuclear extract from 416B haematopoietic progenitor cells resulted in a clear band shift that was out-competed by excess unlabelled wild type probe, but not by probe mutated at the proposed CTCF binding site. In addition, binding of the complex was super-shifted by the addition of anti-CTCF antibody (Figure 3B). To confirm whether CTCF binds the +45 element *in vivo*, ChIP-on-chip assays were performed with a CTCF antibody in 416B and mES cells. Representative plots are shown in figure 3C. In both cell types the most prominent enrichment with CTCF was observed at the +45 element. Enrichment was also observed at the Scl and Map17 promoters in 416B, similar to what was observed in human K562 cells [31]. Analysis of publicly available CTCF ChIP-seq data from the ENCODE consortium [27] clearly demonstrates CTCF binding to the +45 element across a wide range of mouse tissues consistent with the presence of a ubiquitous CTCF site (Figure S4A in Information S1). Since sequence conservation outside of the CTCF binding motif is only moderate between human and mouse, we confirmed that the corresponding human element is also bound by CTCF in a range of human cell lines (Figure S4B in Information S1) using available data from the ENCODE consortium [27]. Together these data strongly suggest that a CTCF-containing protein complex binds to the core 57 base pair region of the +45 element in diverse cell type irrespective of Scl/Map17 expression.

### The 1.76 kb +45 element and the smaller core sequences function as insulators in enhancer blocking assays

We next proceeded to investigate potential insulator function of the +45 element by inserting it into a construct designed to assess enhancer-blocking activity in K562 cells. The construct was used to assess whether DNA placed between an upstream enhancer and a neomycin resistance gene could function as an enhancer-blocker and prevent expression of the resistance gene [32].

**Figure 4:**
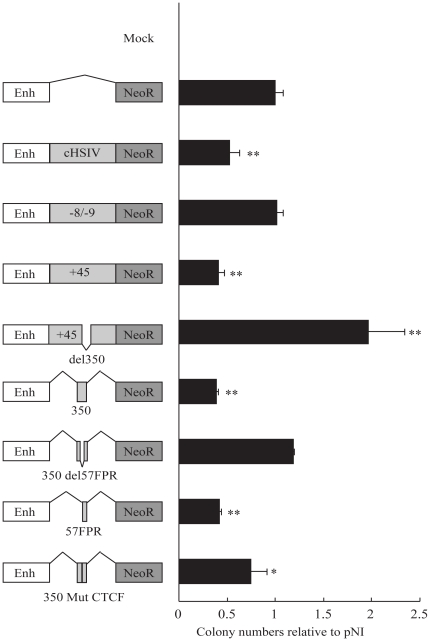
The +45 element function as an insulator and this activity is dependent on the 57FPR. The left panel represents the different constructs diagrammatically. Data is presented as K562 colony numbers relative to the control pNI construct containing only the ß-globin HS2 enhancer and the neomycin resistance promoter. The positive control (1.2 kb fragment containing the chicken HSIV insulator) reduces the number of colonies by 50%, with similar insulation observed from the 1.76 kb +45 element containing the 350 bp core element and the 57FPR. Deletion of either the 350 bp core from the +45 or deletion of the 57FPR from the 350 bp core, both removed the insulator function of the elements. Furthermore, mutation of the CTCF-binding site in the 350 bp core element significantly reduced the insulator function of the 350 bp core element. The data represents the mean ±SD of relative colony numbers from 3 independent experiments each performed in triplicate. * p<0.05, ** p<0.01 when compared to pNI.

As shown in Figure 4, the 1.2 kb fragment containing the chicken beta-globin HSIV (cHSIV) insulator acted as a positive control, approximately halving the number of drug-resistant colonies when placed between the upstream enhancer and the neomycin resistance gene. Similarly, the +45 element had clear insulator activity, decreasing colony numbers by over 50% (Figure 4, compare construct cHSIV with +45), whereas a negative control 2 kb fragment of the Scl locus taken from 8 to 10 kb 5′ of the Scl promoter 1a had no effect on colony numbers. Insulator function was lost when the central 350 bp of the +45 element was removed, while the 350 bp region on its own was as effective as the complete +45 element. Furthermore, deleting the 57 bp footprinted region from the 350 bp element completely abolished insulator function, and the 57 bp sequence on its own exhibited insulator function equivalent to either the 350 bp core or the entire 1.76 kb +45 element. Mutation of the CTCF site removed much of the insulator capacity of the 350 bp central region. Together these data illustrate that the 57 bp footprinted region is both necessary and sufficient for insulator function of the +45 element.

### The 350 bp core region insulates transgenic constructs and improves tissue-specific expression from the Scl promoter

**Figure 5:**
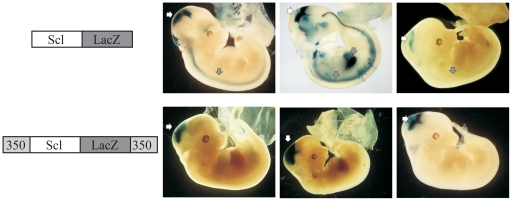
The 350 kb core region of the +45 element improves tissue-specific expression of transgenes. The 350/Scl/LacZ/350 transgenic embryos consistently expressed LacZ in mid and hind brain (white arrow), reflecting endogenous expression pattern of Scl in brain, Scl/LacZ transgenic embryos show ectopic expression (grey arrow). The transgenic constructs are depicted diagrammatically on the left panel, with photographs of 3 representative transgenic embryos presented on the right.

To corroborate the results of our *in vitro* enhancer-blocking assay, we developed an *in vivo* transgenic reporter assay for insulator function. Extensive transgenic analysis by us and others has shown that Scl promoter fragments [6], [15] as well as Map17 promoter fragments (data not shown) do not possess haematopoietic activity in transgenic mice. Therefore, individual candidate elements were assessed for their effect on the efficiency and specificity of expression from LacZ reporter cassettes under the control of the Scl promoter, which alone drives expression exclusively in specific regions of the midbrain and hindbrain [15]. Day 12.5 transgenic embryos were assessed for LacZ expression and the results are shown in Table 1 and Figure 5. The presence of the putative insulator sequences increased the expression efficiency of the LacZ reporter gene in transgenic embryos from 38% (5/13) to 66% (8/12). In the absence of the 350 bp region, the Scl/LacZ constructs gave rise to ectopic expression in 4/5 embryos, whereas, in the presence of flanking 350 bp fragments, ectopic expression (*i.e*, in any other tissue other than brain) was not seen in any of 8 LacZ expressing embryos (p<0.01). These data demonstrate that the presence of flanking 350 bp fragments increased the proportion of LacZ expressing embryos as well as significantly increased the tissue-specific expression from the Scl promoter *in vivo*. This is consistent with the barrier function of an insulator, by preventing position effect variegation due to integration site of the transgene.

## Discussion

We have used array-based DNaseI hypersensitive site mapping to identify a novel regulatory element 45 kb downstream of the mouse Scl promoter 1a. We have shown that this element binds CTCF using both *in vivo* and *in vitro* assays, and that protein-DNA contacts extend to 57 base pairs around a CTCF-consensus binding site. Furthermore, we have shown that this element functions as an insulator in enhancer-blocking assays when tested in haematopoietic cell lines and we have used a novel transgenic reporter assay to demonstrate that the element can both enhance the efficiency and specificity of midbrain-specific expression compared to conventional transgenic analysis. Moreover, analysis of genome-wide data from the ENCODE consortium confirmed that the +45 element is a ubiquitous CTCF-bound insulator region. This element is identified as a DNaseI HSS bound by CTCF in a variety of Scl/Map17 expressing and non-expressing tissues, including haematopoietic tissues (bone marrow and spleen), brain, kidney, liver, heart and lung.

The spatial location of this Scl insulator in relation to neighbouring genes has clear similarities with other recently identified insulator sequences. Mapping genome-wide DNase I sites and chromatin structural elements has confirmed that many genes appear to be flanked by HSS that appear ubiquitous in a range of cell types. Data from high throughput protocols have shown that 86% of ubiquitous DNaseI HSS are within 2 kb of a transcription start site (TSS), and of the remaining ubiquitous DNase HSS which are distal to TSS, over 70% have been shown to bind CTCF in ChIP-chip assays. Furthermore, analysis of the recently identified CTCF consensus binding site, shared by this Scl insulator, has shown that more than 80% of evolutionary conserved sites are greater than 10 kb distal to a TSS. In this study, we have used DNase-Chip technology to show that the +45 element is a DNase HSS in both Scl-expressing and Scl-non-expressing cells, and using a range of *in vitro* and *in vivo* assays, we have shown that it has CTCF-dependent insulator function. ChIP-chip assays for CTCF in human cells have now also identified the equivalent human sequence as a CTCF binding site [31].

Several insulators have been identified to date, the chicken 5′ HSIV being the first identified in vertebrates [33] and by far the best characterized. This element possesses both enhancer-blocking and barrier activity, the hallmarks of an insulator element. Whereas the enhancer-blocking activity is exclusively dependent of CTCF binding, its barrier activity is dependent of the binding of transcription factors like USF1 and USF2 [34]. A second insulator has been identified at the 3′ end of the chicken ß-globin locus, consisting exclusively of a CTCF binding site and possessing exclusively enhancer-blocking activity [35]. The different functions of these two insulators reflect the status of the adjacent chromatin. The presence of heterochromatin 5′ of the chicken ß-globin locus requires a boundary element to prevent it from spreading over the locus. The olfactory receptors present 3′ of the ß-globin genes are not expressed in erythroid cells but are expressed in other tissues, therefore the 3′ insulator only need to possess enhancer-blocking activity. Several other insulators with exclusively enhancer-blocking activity have been identified like Igf2/H19 ICM which contains four CTCF binding sites [36], [37]. To investigate which type of function is performed by the +45 element we perform ChIP-chip for different histone modifications characteristic of active (H3K9Ac) and repressive (H3K27me3) chromatin. In expressing cells (416B) the Scl/Map17 locus is clearly marked by H3K9Ac, not present over the Cyp4X1, while in non-expressed cells it is marked by the repressive mark H3K27me3. These experiments indicate that the +45 element is located at the boundary between chromatin domains, where the Scl/Map17 co-regulatory domain abuts the Cyp gene family.

Until recently, there has been no clear consensus for the genomic sequence of the CTCF transcription factor-binding site. However, using high throughput techniques a new consensus CTCF sequence has been proposed. Intriguingly, a number of well-characterised insulators such as the chicken HSIV, the mouse ß-globin HS2 and the human myb HS2 have relatively little sequence similarity [38], and display only partial similarities to the newly identified consensus sequence. In contrast, the +45 CTCF site that we have functionally mapped in this study shows tight sequence conservation with the new CTCF-consensus sequence, yet was not identified through CTCF binding site consensus mapping. This study therefore provides an independent functional verification of the new CTCF consensus sequence.

The CTCF protein is a large 11 zinc-finger complex that has been shown to contact DNA over many base pairs at other previously-mapped insulator elements [39]. In this study we have, for the first time, used *in vivo* DMS footprinting to map the extent of the protein-DNA interactions at a CTCF-dependent insulator. This shows that *in vivo*, the protein-DNA interaction extends to 57 base pairs of DNA, i.e. beyond the CTCF-consensus binding sequence, suggesting that the structure of DNA surrounding the consensus binding site is likely to play a key role in the CTCF protein-DNA interaction. Moreover we demonstrate that the 57 bp core fragment is both necessary and sufficient for insulator function *in vitro*.

In order to further dissect the +45 insulator function we performed the classic enhancer-blocking assay in haematopoietic cell lines. Introduction of the full +45 element, the 350 bp core region or the 57FPR between the enhancer and the Neo reporter gene lead to a reduction in Neo resistant colonies, similarly to the 1.2 kb cHSIV insulator. The number of colonies was unaltered or even increased when the CTCF binding site in the +45 element was deleted or mutated (del350, 350del57FPR and 350mutCTCF) indicating the CTCF-dependent enhancer-blocking activity. This may be explained by the fact that different cell lines were used for these assays (murine 416B and human K562). To check the insulator function of the +45 element *in vivo*, we elected to develop a transgenic mouse reporter model of insulator function. Using this approach we have demonstrated that the +45 insulator can be used to improve both the frequency of transgene expression (i.e. the proportion of transgenic embryos which express LacZ) and reduces ectopic transgene expression. The Scl promoters drive expression to the brain but not to haematopoietic tissues [15]. Flanking the Scl promoter driven Lacz reporter gene cassette with the +45 element very significantly reduced reporter gene expression in tissues other than brain, the staining pattern expected for the Scl promoter. The discrepancy of tissue types used for the *in vitro* (haematopoietic) and *in vivo* (brain) assays was necessitated by the restricted *in vivo* activity of Scl and Map17 promoters, none of which drive expression in haematopoietic cells *in viv*o when assayed in transgenic mice. Taken together, these data raise the possibility that the +45 element could be a useful tool allowing effective insulation of transgenic constructs from adjacent chromatin.

In conclusion, we have presented data that identifies and functionally defines a putative novel insulator sequence located 45 kb upstream of the Scl transcription start site at the boundary of chromatin domains. Our results suggest that the +45 element functions as a CTCF-dependent insulator, that may prevent inappropriate activation both of the Cyp genes during haematopoietic differentiation and also of Scl and Map17 during hepatic development.

## Supporting Information

Information S1 **Information S1 materials for the manuscript “Mapping and Functional Characterisation of a CTCF-dependent Insulator Element at the 3′ Border of the murine Scl Transcriptional Domain” by Follows et al, including Supporting Figures S1 to S4.**
(PDF)
