# Supplementary material for: Mapping and Functional Characterisation of a CTCF-Dependent Insulator Element at the 3′ Border of the Murine Scl Transcriptional Domain
Source: PLoS One. 2012 Mar 1;7(3):e31484. doi: 10.1371/journal.pone.0031484 (PMC3291548; doi:10.1371/journal.pone.0031484)
Supplement: Information S1 — materials for the manuscript “Mapping and Functional Characterisation of a CTCF-dependent Insulator Element at the 3′ Border of the murine Scl Transcriptional Domain” by Follows et al, including Supporting Figures S1 to S4. (PDF) [file pone.0031484.s001.pdf]

A.

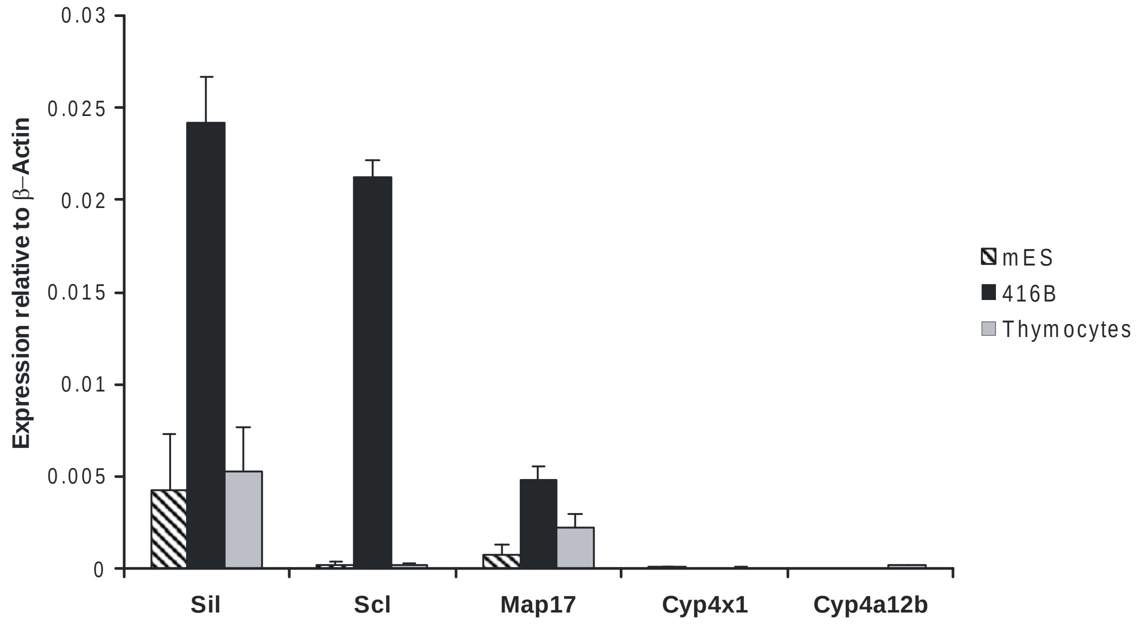

B.

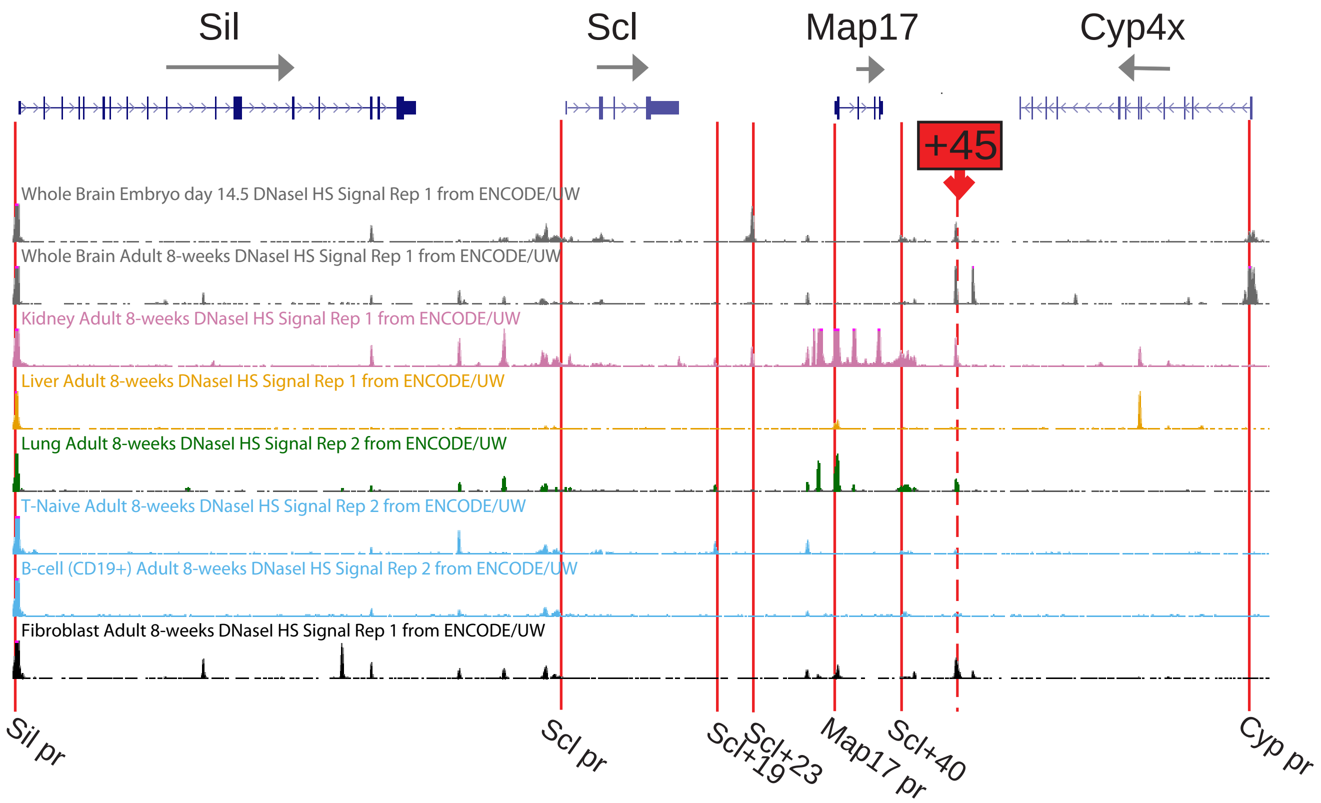

Figure S1. (A) mRNA expression of Sil, Scl, Map17, Cyp4x1 and Cyp4a12b in 416B cells, mES cells and primary mouse thymocytes. Quantitative expression was calculated as relative to β-actin expression using control amplification from plasmid constructs as previously described. The data represents the mean of 2 independent RT-PCR experiments performed in triplicate from independent biological replicates. **(B)** Snap shot of the UCSC genome browser for the mm9 preview assembly showing DNaseI Hypersensitivity profile across the Scl/Map17 locus in a range of mouse tissues from ENCODE/University of Washington. The location of the 4 genes at the locus are represented at the top of the figure (Sil, Scl, Map17 and Cyp4x) with direction of transcription indicated by the arrows and translated and untranslated exons represented by wide and narrow bars respectively. The location of known key regulatory elements is indicated by the red bars (Sil promoter, Scl promoter, Scl+19, Scl+23, Map17 promoter, Scl+40, and Cyp promoter), and the location of the putative +45 element is indicated with a dashed red line.

```
                                                                    NsiI
mouse : GTAC-TCAGTACCAAAGTATGCAAACTAGAGCAAAATAAATATGCATTCTATGAGGTTGTGGCAATCAATAAAAAGAG :
human : GTATATCAGGGACTGAATATGCAAATTAGGGATAAAATGAGGCCCATCTTATGAGGTTGTGGTGAAGAGTAAATGGA- :

mouse : TTATCCTGTCTTCAGAAAGCCTGCAGC-TACTGAACTTTAACAACTGCCCCTCCAGGTTTTGCTTAGCTGTCTTGTCT :
human : --------TGTAAAGAAAGCATGCAGTATGCTCAG--TGAATGACAGCCA-TCGTGATTTTCCT----TTCCTTGCCT :

mouse : GCAATGCCTATAGTGGCCACTAGATGGCCACACCGTGCTTCAGCTTTCTTCCTTGGCAGCGGCTTCTCTCCCACACAG :
human : GCAATATCTCGAGCAGCCACTAGATGACTCCACCT--------CCTTCTCCCTTTCCAGTG--CTGCCAACACTACTG :

mouse : CAATGAGGCTTTTCCACCTCCCCTCCCCTCAGTGCTGTGCCAACTATGCTTAGGATTTCTGGTTTTCCTTCTGTGGG :
human : CTG-GAACTTCTGCCTCCCCTCCCCTCCCTCACCACACATCTCCATGCCCGTGTTGTTCATGTTTTTCCTTCTGCTAG :

mouse : CCTCACCACCTCCAGGAAGGTTCTCTGACATCTCTTTGCATACTACATTCATCAGGTGGACCGAGCTCCGGCCTGTGA :
human : CCTCACCGTCTTCGGGGCGTCTCCCTGACATCTTTT-----ACTACAT------------CTCACCCTCAGCTCTTGG :

        EcoRI
mouse : ATTCCTGGAGGGCCGAGCATAGTGGAATGCAGGCGAAGTGCCTGCATAGTGGAAGTGCTCCACATTTGTTGACCGACG :
human : AGGACGGGATGTCCCCAC------GAAAACTGGCATAGTGCCTGCATTGAACAAGTACTCAGTGTTTGCTGAATGA-A :
```

Figure S2. A manual sequence alignment of the mouse and human central section of the +45 element, showing extensive sequence conservation over the region defined as the 350 base pair core region (located at chr4: 114774278 – 114774628), flanked by the restriction enzyme sites NsiI and EcoRI indicated in the figure.

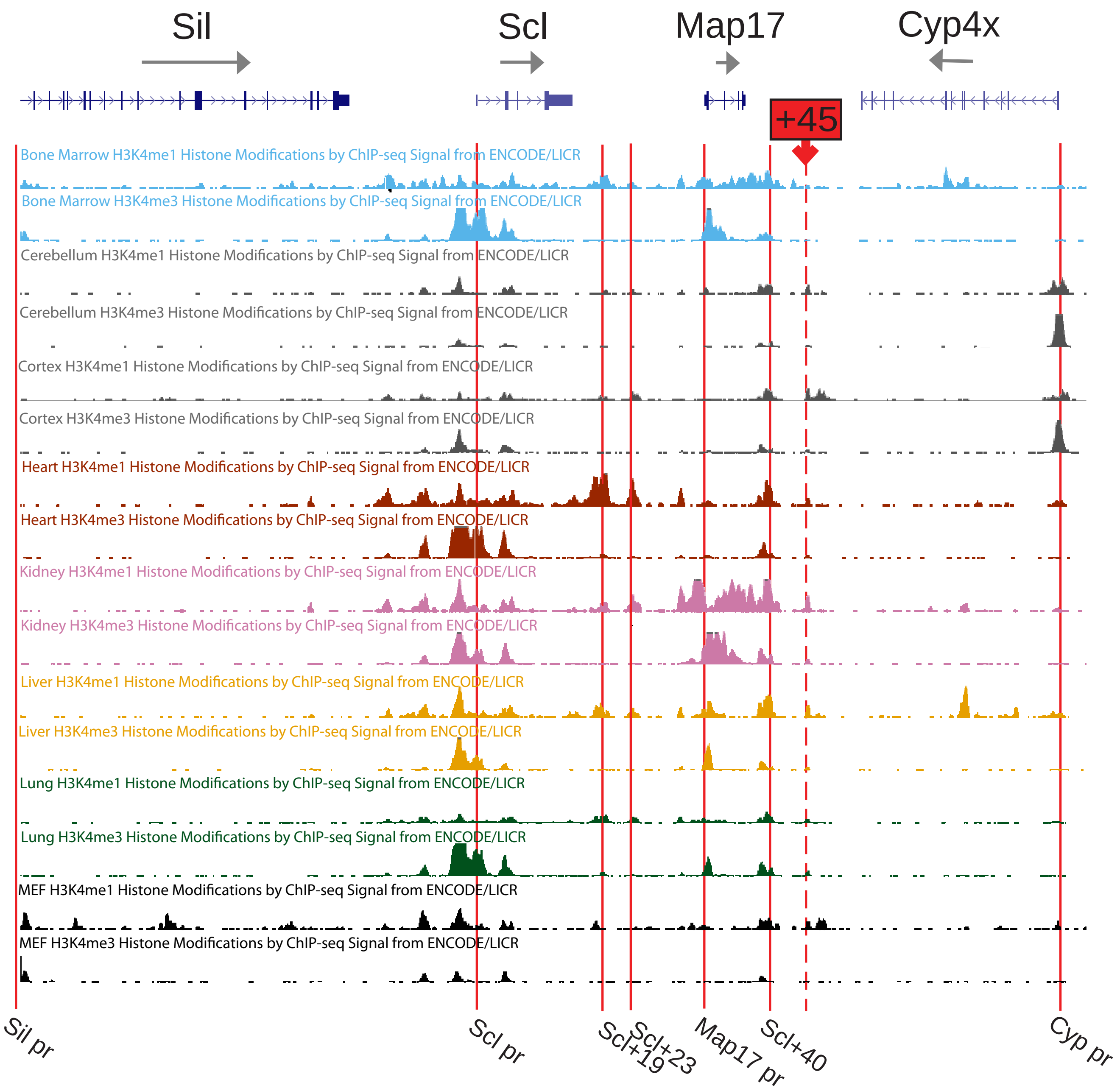

Figure S3. Snap shot of the UCSC genome browser for the mm9 preview assembly showing H3K4me1 and H3K4me3 Histone Modifications by ChIP-seq across the Scl/Map17 locus from ENCODE/LICR, in a range of mouse tissues. The location of the 4 genes at the locus are represented at the top of the figure (Sil, Scl, Map17 and Cyp4x) with direction of transcription indicated by the arrows and translated and untranslated exons represented by wide and narrow bars respectively. The location of known key regulatory elements is indicated by the red bars (Sil promoter, Scl promoter, Scl+19, Scl+23, Map17 promoter, Scl+40, and Cyp promoter), and the location of the putative +45 element is indicated with a dashed red line.

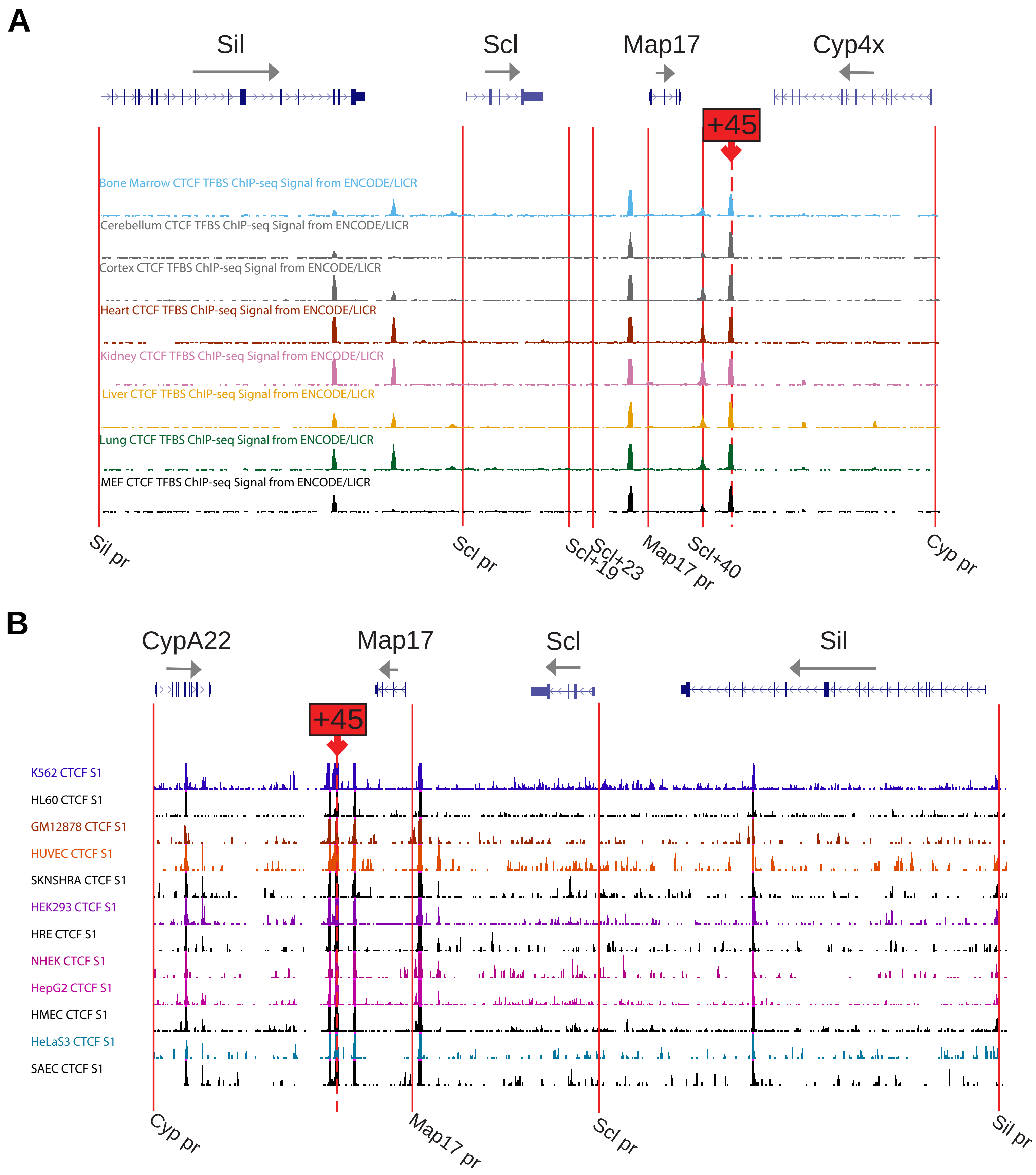

Figure S4. Snap shot of the UCSC genome browser for the mm9 **(A)** and hg18 **(B)** preview assembly showing CTCF transcription factor binding sites by chip-seq from ENCODE, in a range of mouse tissues **(A)** and human cell lines **(B)**. The location of the 4 genes at the locus are represented at the top of the figure (Sil, Scl, Map17 and Cyp4x/Cyp4A2) with direction of transcription indicated by the arrows and translated and untranslated exons represented by wide and narrow bars respectively. The location of known key regulatory elements is indicated by the red bars (Sil promoter, Scl promoter, Map17 promoter, and Cyp promoter), and the location of the putative +45 element is indicated with a dashed red line.
